# Role of Vitamin D in Systemic Sclerosis: A Systematic Literature Review

**DOI:** 10.1155/2021/9782994

**Published:** 2021-11-29

**Authors:** Alexandra-Diana Diaconu, Iustina Ostafie, Alexandr Ceasovschih, Victorița Șorodoc, Cătălina Lionte, Codrina Ancuța, Laurențiu Șorodoc

**Affiliations:** ^1^2nd Rheumatology Department, Clinical Rehabilitation Hospital, 700661 Iasi, Romania; ^2^2nd Internal Medicine Department, “Sf. Spiridon” Emergency Clinic County Hospital, 700111 Iasi, Romania; ^3^Faculty of Medicine, University of Medicine and Pharmacy “Grigore T. Popa”, 700115 Iasi, Romania

## Abstract

**Background:**

Systemic sclerosis (SSc) is a chronic multisystem autoimmune condition defined by a complex pathobiology, comprising excessive fibrosis of skin and internal organs, peripheral vasculopathy with endothelial cell dysfunction, inadequate vascular repair and neovascularization, and aberrant immunity. Vitamin D is a steroid hormone with pleiotropic effects beyond its traditional role in calcium and bone homeostasis. Since vitamin D has immunomodulatory, cardioprotective, and antifibrotic properties, it could potentially interfere with SSc pathogenesis. Suboptimal vitamin D levels are classically recognized in scleroderma, irrespective of clinical and serological phenotype.

**Aim:**

This systematic review is aimed at investigating and clarifying the role of vitamin D in SSc and emphasizing the association of vitamin D status with different clinical settings.

**Methods and Results:**

A systematic online search was performed, using PubMed databases to collect articles on the topic of vitamin D in SSc. The final analysis included 40 eligible articles.

**Conclusions:**

Hypovitaminosis D is common in SSc patients and could be associated with clinical and serologic patterns of the disease. Intervention for low serum vitamin D levels in SSc pathogenesis remains controversial, as well as the significance of vitamin D supplementation in such patients.

## 1. Introduction

### 1.1. Systemic Sclerosis

Classified as an orphan disease, systemic sclerosis (SSc) remains a chronic multisystem autoimmune condition driven by a multifaceted link between excessive fibrosis of the skin and internal organs, peripheral vasculopathy with endothelial cell dysfunction succeeded by inadequate vascular repair and neovascularization, and aberrant immunity. The clinical picture ranges from peripheral vasculopathy (Raynaud's phenomenon, digital ulcers, and critical digital ischemia) and skin involvement, to a broad spectrum of manifestations depending on the presence and degree of internal organ (gastrointestinal, lung, kidney, and heart) involvement, resulting in specific phenotypes characterized by precise antibodies, distinct prognoses, and specific management [1, 2].

Although the complex pathobiology of SSc is still not well understood, immunological, vascular, and fibrotic abnormalities remain key events, including (i) fibroproliferative vascular injuries of small arteries and arterioles; (ii) increased production of profibrotic growth factors such as transformer-*β* growth factor (TGF-*β*), connective tissue growth factor, and insulin-like growth factor, leading to fibrosis of the skin and various internal organs; and (iii) various modifications of innate, humoral, and cellular immunity, promoting immune cell trafficking into the affected tissues and autoantibody synthesis. Additionally, the intervention of environmental factors in a genetically susceptible host triggers the autoimmune mechanism specific to SSc [2, 3].

### 1.2. Vitamin D

Vitamin D is a steroid hormone obtained from endogenous release in the skin following sunlight exposure and from food intake. Calcitriol or vitamin D_3_ is actually formed by the activation of a precursor on skin exposed to ultraviolet B radiation and, to become active, requires two metabolic conversions: 25-*α* hydroxylation in the liver and 1-*α* hydroxylation in the kidney [4].

Apart from its traditional role in maintaining calcium homeostasis and bone health [5], vitamin D has pleiotropic effects based on the ubiquitous distribution of vitamin D receptors (VDRs). Belonging to the nuclear hormone receptors superfamily [6], VDRs are able to mediate the multifaceted biological effects of hormone D, as they are found not only in the osteoblasts, small intestine, and kidneys but also in a variety of immune cells (such as activated T and B lymphocytes, mononuclear cells, antigen-presenting cells, and natural killer cells), islet beta cells, and in some organs (such as the heart, brain, skin, breasts, gonads, and prostate) [5]. Beyond the classic antirachitic role, vitamin D has protective effects in many dermatological, cardiovascular, gastrointestinal, pulmonary, renal, and autoimmune disorders [7].

Among the physiological vitamin D actions, the regulation of cell differentiation, proliferation, apoptosis, angiogenesis, innate or adaptive immunity, and immune modulation are of particular interest in immune-mediated rheumatic disorders. Vitamin D mainly exerts its immunosuppressive effects by inhibiting T helper-1 (Th1) lymphocytes and proinflammatory cytokines (IL-6 and IL-17) and stimulating anti-inflammatory cytokine production (IL-4 and IL-10). Furthermore, it is able to switch the Th1 response (IL-1, TNF-*α*, and IFN-*γ*) into a Th2 one (autoantibodies and TGF-*β*). Vitamin D also hampers the Th17 response and stimulates regulatory T (Treg) cells. Therefore, vitamin D seems to be protective in the development of autoimmunity, related to its immunomodulatory and tolerance effects [7].

Interestingly, vitamin D has antifibrotic properties, being able to modulate TGF-*β* signaling and inhibit the profibrotic phenotype of skin and lung fibroblasts. In fact, vitamin D downregulates the expression of profibrotic factors such as TGF-*β*1 and collagen I and III, while upregulating several antifibrotic factors [7].

Moreover, the role of vitamin D in cardioprotection is widely accepted, although its mechanisms are still poorly understood. It seems that vitamin D acts via the renin system to decrease blood pressure, increase vascular endothelial growth factor, and decrease the production of endothelium-derived contracting factors [7].

The Institute of Medicine (Washington DC) has proposed cut-off values for normal plasma vitamin D levels (>30 ng/ml), vitamin D insufficiency (21–30 ng/ml), and deficiency (<20 ng/ml) [8]. While vitamin D deficiency results in impaired phospho-calcium homeostasis and promotes low bone mineral density and osteoporosis [9], poor vitamin D status may also trigger various autoimmune conditions [7].

### 1.3. Vitamin D in Systemic Sclerosis

In the last decades, many authors have focused on vitamin D status in systemic sclerosis. Due to its immunomodulatory, cardioprotective, and antifibrotic biological effects, vitamin D could interfere with each of the pathobiological mechanisms activated in SSc, including autoimmunity, peripheral vasculopathy, and fibrosis. A majority of studies have emphasized suboptimal vitamin D levels in SSc patients, irrespective of the clinical or serological phenotype. It seems that vitamin D levels are significantly lower compared to healthy controls, and vitamin D supplementation at the usual dose fails to correct the deficit. Furthermore, due to the extreme clinical heterogeneity of the disease, poor vitamin D status in SSc may or may not be associated with different systemic manifestations of the disease, SSc activity and severity, or disease subtype [7].

Despite extensive research on vitamin D in autoimmune conditions, the potential role of hypovitaminosis D in the pathobiology of scleroderma, the dual relationship between serum vitamin D levels and different clinical manifestations, and the importance of vitamin D supplementation within the clinical and serologic spectrum of SSc remain controversial.

This systematic literature review is aimed at investigating the pathobiological role of vitamin D in systemic sclerosis and emphasizing the association of vitamin D status with different clinical settings.

## 2. Methods

A systematic online search was performed using the PubMed databases up to 31 May 2021 to collect articles on vitamin D in systemic sclerosis, without any criteria based on the year, language, or type of publication. We used the following combinations of terms: “systemic sclerosis” and “vitamin D”, “scleroderma” and “vitamin D”, and “systemic scleroderma” and “vitamin D”.

All articles were retrieved and assessed independently by two reviewers, who highlighted data regarding the authors, publication date, and characteristics of the study. After rejecting abstracts and duplicates, all articles in full text were examined to verify their eligibility criteria, which included studies on the association between vitamin D and SSc. The exclusion criteria were preprint papers, articles with no relevant content to the purpose of the research, reviews, studies with a pediatric population, *in vitro* studies or experimental models, and studies with a small group of patients (case series and case reports).

## 3. Results

**Figure 1:**
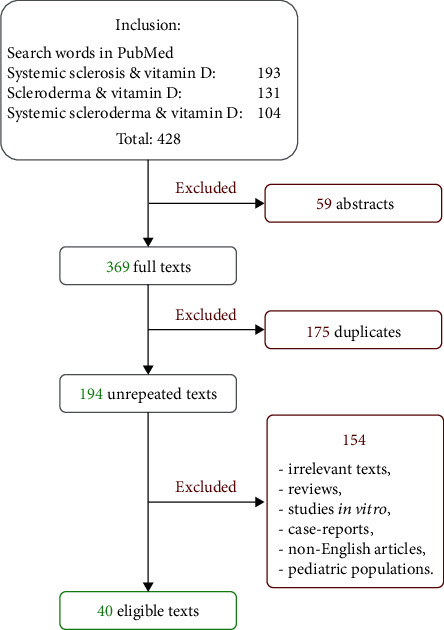
Flowchart of study selection.

We identified 428 publications focusing on vitamin D in SSc in the PubMed databases. The final analysis included 40 eligible articles after 388 were excluded (59 abstracts, 175 duplicates, 154 irrelevant, *in vitro* experimental models on the vitamin D effects or its analogues, case reports, non-English, or pediatric population articles). These data are illustrated in Figure 1.

Study design, number of cases, and main outcomes of selected articles are summarized in Table 1. The majority of studies focused on assessing vitamin D levels in SSc and the relationship between vitamin D and different disease subtypes and clinical and serologic scenarios, while only a few articles focused on potential pathobiological relationships or the role of vitamin D supplementation.

## 4. Discussions

### 4.1. Hypovitaminosis D in SSc

Vitamin D deficiency has been implicated in many degenerative, metabolic, inflammatory, and autoimmune rheumatic conditions, including systemic sclerosis. The prevalence of hypovitaminosis D in SSc remains a topic of interest, and several papers have aimed to investigate the association between serum vitamin D levels and different aspects of SSc. Overall, irrespective of the clinical and serological disease settings, SSc patients present with lower levels of vitamin D compared with healthy controls [12, 14, 15, 19, 20, 23–26, 31, 35, 37, 44, 45]. Although hypovitaminosis D is frequently reported, some authors have found significantly lower levels (*p* < 0.001) and vitamin D deficiency [15, 19, 20, 25, 31, 35, 37], while others have found only insufficient levels. A closer look at the literature confirmed lower vitamin D levels in patients with SSc, independent of the well-known age, gender, and seasonal variations, as well as related to comorbidities, drugs, and life habits [14, 15, 19, 20, 23–26, 31, 35, 37, 40, 41, 45].

Although seasonal variations in vitamin D levels are predictable, with peak values during summer [22], Seriolo et al. found lower average serum 25(OH)D concentrations in all seasons in SSc patients [42].

Additionally, in a retrospective cohort study, Arnson et al. found a negative correlation between vitamin D concentrations and age (*p* < 0.05) [40], while many other studies were unable to demonstrate any correlation between serum vitamin D and age, gender, body mass index (BMI), and therapy in SSc [20, 22, 26, 29, 32, 33, 41, 42]. Moreover, there is no association between vitamin D serum levels and the duration and frequency of sun exposure or sunblock use in SSc [22].

Conversely, Sampaio-Barros et al. demonstrated a positive correlation between vitamin D levels and weight and BMI [32], and Arnson et al. found a negative correlation between vitamin D concentration and patient age in SSc [40]. Only one study [46] emphasized the association between hypovitaminosis D and ethnicity (Arab origin) in SSc.

Furthermore, the relationship between disease duration and hypovitaminosis D also appears to be variable, probably because of the heterogeneity of the populations investigated. In a study of 65 SSc patients, Caramaschi et al. found an association between vitamin D deficiency and longer disease duration.

Although the role of vitamin D in the development of different clinical and serological phenotypes of SSc remains controversial, it has been suggested that hypovitaminosis D may have a role in disease activity and/or severity. However, this hypothesis was not validated in our review; the majority of authors demonstrated no association between vitamin D levels and disease activity [16, 19, 21, 22, 33, 34, 46] or severity (Medsger disease severity scores) [6, 33]. Only Vacca et al. emphasized a significant negative correlation between vitamin D and the European Disease Activity Score [6].

Another important point is related to the true origin of low-serum vitamin D in SSc. Poor vitamin D status in SSc could be multifactorial, since its normal metabolism typically comprises epidermal synthesis, gastrointestinal absorption, and hepatic and renal hydroxylation steps, and SSc is defined by various degrees of skin, gastrointestinal, and renal involvement. Though still controversial, potential explanations for either insufficient or deficient vitamin D include (i) impaired epidermal synthesis due to skin thickening and hyperpigmentation; (ii) insufficient intake or impaired gastrointestinal absorption due to gastrointestinal involvement and/or certain drugs (such as glucocorticoids or anticonvulsants for neuropathic pain) that could interfere with vitamin absorption; and (iii) limited sun exposure related to physical disability, depression, and potential psychological limitations [30]. Moreover, according to Carmel et al., the presence of vitamin D antibodies may also account for lower vitamin D levels in SSc, as they found anti-25(OH) vitamin D IgM antibodies more frequently in SSc cases compared to controls [33].

Some studies have focused on statistically significant relationships between vitamin D and parathyroid hormone (PTH), not only in healthy individuals but also in SSc patients [12, 13, 31, 35, 46]. Furthermore, Hax et al. demonstrated that 25-hydroxyvitamin D [25(OH)D] levels are inversely correlated with PTH [12]. Paolino et al. identified significant differences in malnourished SSc patients in the median values of PTH and vitamin D levels [13].

### 4.2. Vitamin D Levels and SSc-Related Clinical and Serologic Manifestations

#### 4.2.1. Skin Involvement and Clinical Phenotype

Almost always involved in systemic sclerosis, skin is a marker of disease activity, severity, and prognosis. The modified Rodnan skin score (mRSS) remains the most appropriate technique to evaluate the extent and severity of skin fibrosis [49]. Extensive and excessive skin thickening, as well as widespread skin fibrosis, correlates with severe internal organ involvement, high disability, and poor prognosis [49].

It has been suggested that typical skin manifestations such as fibrosis and, to a lesser extent, calcinosis cutis may reduce vitamin D synthesis and influence the other clinical manifestations in scleroderma patients, with differences between diffuse (dcSSc) and limited cutaneous SSc (lcSSc). A closer look at the potential association between levels of vitamin D and the clinical phenotype of SSc showed no significant difference in vitamin D or its active metabolite 25(OH)D levels, irrespective of the disease subtype, in the majority of studies [10, 16, 22–24, 41, 45]. However, An et al. demonstrated lower serum vitamin D in SSc compared with healthy controls and in dcSSc compared to lcSSc. Further, Corrado et al. found significantly lower 25(OH)D levels in dcSSc compared to lcSSc.

Lower serum vitamin D inversely correlates with the extent of cutaneous fibrosis, as reported by two studies [31, 40]. In their retrospective cohort study including 327 SSc patients, Arnson et al. demonstrated an inverse correlation between skin fibrosis and vitamin D levels [40]. Additionally, Atteritano et al. showed higher mRSS in SSc with vitamin D insufficiency [31]. On the other hand, several papers failed to report associations between vitamin D concentrations and the extent of skin involvement or mRSS [16, 21, 41, 48].

It is widely recognized that vitamin D also has antifibrotic properties related to its essential intervention in fibrogenesis through the modulation of TGF-*β* activity. Moreover, the inhibitory effects of vitamin D on collagen and hyaluronate production induced by TGF-*β* have been extensively recognized. TGF-*β* remains a key factor involved in collagen synthesis by fibroblasts, and VDR expression is a negative regulator of the TGF-*β*, a signaling pathway. The inhibition of the profibrotic TGF-*β* signaling via vitamin D is enhanced by its role in polarizing the local immune response. In addition, it seems that aberrant VDR expression in fibroblasts in SSc patients is responsible for the profibrotic effects of TGF-*β* on fibroblasts via decreased vitamin D concentrations [20]. This is why lower levels of vitamin D in SSc patients may be related to skin fibrosis, and the correction of hypovitaminosis D could interfere with fibrosis.

Interestingly, Humbert et al. described a significantly improved skin flexibility after vitamin D supplementation [48] but, more recently, Hulshof et al. failed to recognize any significant variation in mRSS after 9 months of vitamin D supplementation in their double-blind placebo-controlled trial [47].

Finally, regarding the association between vitamin D and calcinosis cutis (an SSc cutaneous manifestation commonly found in CREST syndrome), the majority of studies described no significant correlation between 25(OH)D levels and the presence of calcinosis in patients with SSc [6, 25, 27, 29, 41].

#### 4.2.2. Peripheral Digital Vasculopathy

Different studies have investigated the association between low vitamin D levels and peripheral vasculopathy in SSc patients, particularly Raynaud's phenomenon and digital ulcers. Although this is a nonspecific but widely reported vasospastic condition in the majority of patients with scleroderma [50, 51], it seems that neither the severity nor the duration of Raynaud's phenomenon correlates with vitamin D levels [22]. SSc patients with low vitamin D have an increased risk of developing digital ulcers, as supported by multiple studies [16, 28], and vitamin D deficiency may be an independent risk factor for digital ulcers in such patients. Conversely, other papers failed to demonstrate a significant relationship between serum 25(OH)D and digital ulcers [19, 22, 41, 45]. Moreover, in a cross-sectional study, Groseanu et al. pointed out a negative association between vitamin D supplementation and the development of digital ulcers in patients with SSc [30].

Nailfold videocapilaroscopy is largely used to assess microvascular changes in SSc, and specific capillaroscopic patterns not only reflect the diagnosis but also predict internal organ involvement and prognosis [52]. Various different papers have addressed the potential association between vitamin D and capillaroscopic features [32, 43]. Sampaio-Barros et al. clearly showed that diffuse devascularization and avascular areas depicted on videocapillaroscopy are associated with lower vitamin D concentrations in scleroderma patients [32]. Additionally, Caramaschi et al. found an association between the late nailfold videocapillaroscopy pattern and vitamin D deficiency [43].

**Figure 2:**
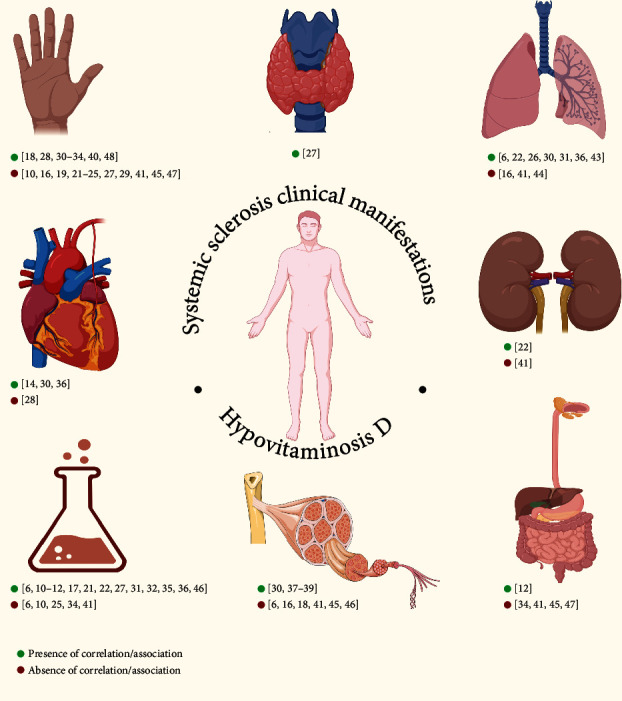
Relationship between systemic sclerosis clinical manifestations and hypovitaminosis D.

Extreme clinical heterogeneity of SSc is closely related to a broad spectrum of internal organ involvement comprising, but not limited to, gastrointestinal, cardiovascular, pulmonary, and renal injuries (Figure 2) [2]. Moreover, SSc can be fatal, with a 3-year survival rate in half of the cases, especially with severe lung or heart involvement [53].

Hypovitaminosis D is a general finding in scleroderma [3–5, 9, 10, 13, 15, 16, 21, 25, 27, 34, 35], and based on its pleiotropic effects, including its immunomodulatory, cardioprotective, and antifibrotic properties, low vitamin D status could influence the pathogenetic pathways activated in SSc [30]. Nevertheless, the association between abnormal vitamin D status and SSc onset, or any of its clinical manifestations, is still under debate [30]. Gupta et al. did not observe any significant association between vitamin D levels and systemic involvement [19]. Conversely, several other studies found that patients with a poor vitamin D status presented with systemic features, particularly lung and cardiac involvement [6, 22, 26, 30, 31, 36, 43].

#### 4.2.3. Pulmonary Involvement

Interstitial lung disease and pulmonary arterial hypertension are the most frequent subtypes of lung involvement (over 80% of cases). Trombetta et al., in a 152 SSc patient retrospective analysis, and Zhang et al., in a case-control study including 120 participants, found a higher rate of pulmonary involvement in patients with vitamin D deficiency [22, 26]. However, data regarding the potential association of vitamin D and pulmonary manifestations in SSc remain controversial. Thus, although Atteritano et al., Caramaschi et al., and Vacca et al. all pointed out a significant correlation between pulmonary hypertension and lower levels of vitamin D [6, 31, 43], Caimmi et al. and Rios Fernández et al. did not find any significant difference between the frequency of pulmonary arterial hypertension and vitamin D concentrations [16, 44]. The exact mechanism and pathways through which suboptimal levels of vitamin D may be responsible for pulmonary hypertension also remain unclear [20]. Potential explanations include the activation of the renin-angiotensin pathway, aberrant expression of prostacyclin by vascular smooth muscle cells, or secondary parathyroidism [30].

It has been suggested that low vitamin D levels may be a factor associated with certain clinical manifestations, especially pulmonary and cardiac involvement in SSc, promoting disease activity and severity. Indeed, hypovitaminosis D significantly correlates with lung involvement [6, 22, 26, 30, 36, 43], with a high ratio of more severe pulmonary fibrosis in SSc patients with vitamin D insufficiency, irrespective of disease subset [26, 30]. A positive correlation between decreased serum vitamin D and pulmonary fibrosis, as well as low diffusion capacity of carbon monoxide (DLCO), has been demonstrated [6, 30, 36]. Moreover, Groseanu et al. confirmed that none of the patients with normal vitamin D status developed lung fibrosis in their study, and lung involvement was reported more frequently in patients with a diffuse form and vitamin D deficiency [30]. On the other hand, Caimmi et al. found an absence of significant differences in vitamin D variations for delta of forced vital capacity, or DLCO, in SSc [16], and An et al. found no correlation between vitamin D deficiency and systolic pulmonary pressure and pulmonary involvement [24]. Rios Fernández et al. and Gambichler et al. failed to support any significant correlation between vitamin D levels and the presence or absence of lung fibrosis [41, 44].

As mentioned previously, vitamin D also acts as a factor influencing the transdifferentiation of lung epithelial cells into myofibroblasts in murine studies, supporting the direct role of vitamin D in fibrosis signaling. This may suggest the potential benefits of using vitamin D as an adjuvant for immunosuppression in patients with clinical findings where fibrosis predominates, such as in lung and skin involvement [30].

#### 4.2.4. Cardiac Involvement

The cardioprotective effect of vitamin D is widely accepted, and significant correlations between vitamin D status and cardiovascular involvement have been reported in scleroderma patients. Since cardiovascular involvement is commonly seen during SSc with repeated focal ischemic lesions, fibroblast replacement, and subsequent irreversible myocardial fibrosis as the main causes of myocardial impact, many authors have been interested in assessing the role of vitamin D deficiency in cardiovascular involvement secondary to SSc. Groseanu et al. were able to demonstrate that patients with SSc and vitamin D deficiency had a higher prevalence of systolic or diastolic dysfunction, as well as rhythm and conduction disorders [30]. Rios Fernández et al. reported an association between vitamin D levels and heart involvement [36].

Vitamin D may also have several effects on microvascular and macrovascular involvement in patients with SSc. Different studies have aimed to clarify the controversial topic of subclinical atherosclerosis in SSc patients by assessing carotid intima-media thickness (cIMT), flow-mediated vasodilatation, and coronary and cerebral calcifications. In a recent study, González-Martín et al. reported an inverse association between serum vitamin D levels and cIMT in SSc [14]. On the other hand, in a pilot study including 120 participants, no differences between the median cIMT, brachial-ankle pulse wave velocity, frequency of carotid plaques, and vitamin D deficiency were demonstrated [28]. Further studies are necessary in order to clarify the association between hypovitaminosis D and micro- and macrovascular involvement in patients with SSc.

#### 4.2.5. Gastrointestinal Tract Involvement

Gastrointestinal involvement has been widely reported in SSc [54, 55], mainly related to vasculopathy and local fibrosis [56] and may potentially prompt vitamin D malabsorption. However, there is no consensus on low vitamin D levels and gastrointestinal manifestations in SSc. The majority of studies have failed to demonstrate any association between gastrointestinal manifestations and suboptimal vitamin D status. Calzolari et al. found no relationship between gastrointestinal involvement and vitamin D concentrations [45]. Moreover, Gambichler et al. documented no association between gastroesophageal reflux disease and vitamin D [41], while Hulshof et al. also revealed no significant differences in esophageal motility after 9 months of vitamin D supplementation [47]. Small intestinal dysmotility caused by atrophy or fibrosis in the intestinal wall seems to be quite common in patients with SSc and may be complicated by bacterial overgrowth and malnutrition [55]. However, Corrado et al. found no correlation between malabsorption and vitamin D levels [34]. No difference in body mass index values depending on the level of vitamin D was demonstrated either [16, 41].

#### 4.2.6. Renal Involvement

Renal diseases in SSc patients may manifest in different clinicopathological settings, and scleroderma renal crisis is the most severe life-threatening complication [57, 58]. In a 154 patient retrospective analysis, Trombetta et al. found a high ratio of kidney involvement at lower vitamin D levels [22], and Groseanu et al. reported scleroderma renal crisis more frequently in patients with vitamin D deficiency in their cohort with SSc [30]. Conversely, Gambichler et al. did not find any significant relationship between renal involvement and hypovitaminosis D, irrespective of SSc subtype [41].

#### 4.2.7. Musculoskeletal Involvement

Musculoskeletal involvement is a common and potentially disabling manifestation in scleroderma patients [30]. Since vitamin D is essential for proper functioning of the musculoskeletal system, several studies have investigated the role of suboptimal vitamin D levels and articular involvement, acroosteolysis, sarcopenia, and myositis during SSc.

While Ibn Yacoub et al. reported a significant correlation between vitamin D levels and severity of joint pain [37], another study showed no significant statistical association between vitamin D and joint involvement [45]. According to Groseanu et al., patients with vitamin D deficiency developed synovitis more frequently than those with vitamin D insufficiency. A negative correlation was also identified between vitamin D status and muscle weakness; patients with muscle weakness presented with lower vitamin D levels compared to those without [30].

Defined by the European Working Group on Sarcopenia in Older People as a progressive loss of muscle mass and strength, with a risk of adverse outcomes such as disability, poor quality of life, and death [59], sarcopenia may develop in different immune-mediated rheumatic conditions due to muscle loss secondary to abnormal proinflammatory status, pain, decreased activity, and glucocorticoid use. Corallo et al. evaluated the presence of sarcopenia in a cohort of 62 SSc patients using two specific parameters, Relative Skeletal Mass Index and Hand Grip Strength. No association between serum vitamin D levels and sarcopenia was found, but the authors highlighted age-related sarcopenia [18].

Although not pathognomonic, but rather highly suggestive for SSc, acroosteolysis is a distinct pattern of bone resorption which affects the distal phalanges, due to repeated vascular injury. Hyperparathyroidism secondary to low vitamin D might also contribute to acroosteolysis, but we found two studies that showed no correlation between vitamin D and acroosteolysis [6, 46].

Many recent articles have tried to clarify the association of low vitamin D levels with bone mineral density (BMD) in SSc and fracture risk in SSc patients, based on the role of vitamin D in calcium-phosphate and bone homeostasis. Overall, decreased bone mineral density (BMD) was reported in the majority of scleroderma patients presenting with low levels of vitamin D (<30 ng/ml) [36–39]. In addition, Shinjo et al. identified a high prevalence of 25(OH)D insufficiency in SSc that correlates with hip BMD (femoral neck and total femur) [39]. A significant correlation between vitamin D levels and BMD in the lumbar spine and femoral neck was described by ibn Yacoub et al. [37], with positive correlations of vitamin D levels and total femur BMD and femoral neck BMD, as reported by Sampaio-Barros et al. [32]. Furthermore, it seems that low levels of vitamin D represent a risk factor for fractures in scleroderma [38]. A detailed look into bone turnover biomarkers showed a significant negative correlation between serum C-telopeptide of type I collagen (CTX) and vitamin D in SSc [45]. In a comparative study with rheumatoid arthritis, Avouac et al. showed a higher prevalence of osteoporosis and fractures in patients with SSc and identified vitamin D concentrations, as well as age as risk factors, for such complications [38].

#### 4.2.8. Quality of Life

The association of vitamin D and physical functioning in scleroderma is also of interest. Several papers have highlighted the relationship between hypovitaminosis D and physical functioning or quality of life, suggesting a role of decreased levels of vitamin D in worse quality of life. Sampaio-Barros et al. revealed a positive correlation for suboptimal vitamin D levels with quality of life questionnaires, including the SF-36-Vitality, SF-36-Social Function, SF-36-Emotional Role, and SF-36-Mental Health in SSc [32]. Moreover, a negative correlation between 25(OH)D and quality of life was demonstrated particularly in dcSSc, based on the HAQ-Reac and HAQ-Grip Strength. Calzolari et al. showed a significant correlation between vitamin D and physical performance score, assessed by the Medical Outcomes Study Short Form-36 (SF-36) questionnaire [45]. On the other hand, Vacca et al. found no association between vitamin D deficiency and the Health Assessment Questionnaire parameters [6].

#### 4.2.9. Comorbidities/Complications of SSc

Other autoimmune diseases, such as primary biliary cirrhosis, secondary Sjögren syndrome, or thyroid diseases (including Hashimoto's thyroiditis and Graves' disease), are more frequent in SSc patients. Hypothyroidism may develop in up to 15% of patients with SSc, especially lcSSc, which is probably related to thyroid fibrosis [60]. Moreover, thyroid dysfunction may also have an important impact on clinical manifestations of SSc. Raynaud's phenomenon is more difficult to control in hypothyroid patients, and pulmonary hypertension can be severely influenced by hemodynamic changes in hypothyroidism [61].

Toki et al. identified 30 autoimmune thyroid disorder patients out of 210 SSc participants and reported hypothyroidism as more common [62]. Of interest, Giuggioli et al. described a strong association between hypovitaminosis D and autoimmune thyroiditis [27].

Periodontitis is one of the most common inflammatory diseases of adults and represents a chronic oral multibacterial infection [63]. Isola et al., in their clinical trial, found a negative association between periodontitis and vitamin D concentrations in SSc patients. Furthermore, the study emphasized lower vitamin D status in individuals with periodontitis and those with periodontitis and SSc, but not in healthy controls [11].

#### 4.2.10. Serology

A specific antibody signature is known to occur in scleroderma patients, with high levels of circulating antinuclear antibodies (ANAs) associated with positivity for anti-centromere, anti-topoisomerase I (anti-SCL-70), and anti-RNA polymerase III antibodies. There is a close relationship between disease phenotype, clinical features, and antibody profile, with phenotypes supporting triple diagnostics, future organ involvement, and prognostic value [53, 64, 65].

The link between abnormal serum vitamin D and serologic specificities in SSc is still under debate. Several studies have reported that lower levels are significantly associated with positive antinuclear antibodies [36], anti-topoisomerase I antibodies [32], and anti-centromere antibodies [6]. In contrast, Gambichler et al. indicated no significant relationship between vitamin D and antinuclear autoantibodies [25, 41]. Moreover, Corrado et al. reported low vitamin D levels are independent of the presence of anti-topoisomerase I and anti-centromere autoantibodies in SSc patients [34].

Much attention has recently been given to CD34+ CD45- endothelial progenitor cells as a marker of early vascular activation and endothelial lesions in SSc, which could potentially be useful for disease activity estimation [66]. In a recent study, Lo Gullo et al. found an inverse correlation between vitamin D levels and CD34+ cell numbers in SSc patients [10].

Furthermore, it seems that vitamin D is able to increase IL-10 secretion by CD4+ T cells and enhances the number of IL-10-producing T regulatory T (Treg) cells, which are known to suppresses the immune response and mediate immune tolerance [7]. Liberto et al. observed a significantly increased production of Treg cells and IL-10 following 1,25(OH) vitamin D treatment, suggesting a beneficial role of vitamin D supplementation in autoimmunity [17]. In a case-control study, Hax et al. demonstrated that IL-2 and IL-4 serum levels were reduced in SSc patients compared with controls; however, no significant correlation between cytokines and serum levels of vitamin D was demonstrated in such patients [12].

There are some limitations of this systematic literature review. Firstly, only PubMed was systematically search; other databases, such as Cochrane Library and Medline/EMBASE, were not included due to access restrictions. However, a high number of overlaps between these databases should have limited the number of potentially missed papers. Secondly, studies with different designs were included—prospective and retrospective, cross-sectional, case-control studies, and one meta-analysis—which may have resulted in bias in conclusions on the true role of vitamin D in the systemic sclerosis pathobiology.

## 5. Conclusions

In conclusion, hypovitaminosis D is common in systemic sclerosis patients, irrespective of the clinical and serological phenotype.

Because of the pleiotropic roles of vitamin D, including its immunomodulatory, cardioprotective, and antifibrotic properties, suboptimal vitamin D could interfere with all major pathobiological SSc pathways. Additionally, poor vitamin D status may be involved in clinical manifestations and SSc evolution, as shown in this literature review. However, details on the interference between low serum vitamin D levels and SSc pathogenesis remain controversial in some aspects, and future studies are needed to clarify this involvement.

If and when vitamin D levels should be monitored during the course of SSc, as well the significance and optimal dose of vitamin D supplementation, remain open questions.

## Figures and Tables

**Table 1:** Characteristics of selected studies on serum vitamin D in systemic sclerosis.

Reference	Study design	Patients	Outcome
Lo Gullo et al., 2021 [10]	Case-control study	36 SSc patients and 36 healthy controls	Correlation between serum vitamin D and CD34+ cell (*p* = 0.05). No correlation between serum vitamin D levels and CRP (*p* = 0.451). No difference in vitamin D levels in dcSSc patients compared to lcSSc patients. No association between vitamin D, body mass index, and endothelial markers in SSc.
Isola et al., 2021 [11]	Clinical trial	35 SSc patients, 36 with periodontitis (PD), 36 with both SSc and periodontitis, and 37 controls	Lower vitamin D values in subjects with PD and SSc plus PD than to SSc and healthy subjects (*p* < 0.001). Negative association between vitamin D levels and PD in SSc (*p* = 0.011). Association between lower vitamin D and CRP (*p* < 0.01).
Hax et al., 2020 [12]	Case-control study	50 SSc patients and 35 healthy nonmatched controls	Lower vitamin D levels in SSc patients (*p* = 0.014). 25-Hydroxyvitamin D [25(OH)D] levels inversely correlated with parathyroid hormone (PTH) levels (*p* = 0.026). No significant associations between vitamin D and serum cytokines. No association between vitamin D serum levels and the duration and frequency of sun exposure (*p* = 0.417 and *p* = 0.295, respectively) or with the sun block use (*p* = 0.857).
Paolino et al., 2020 [13]	Retrospective study	36 consecutive postmenopausal SSc female patients	Significant differences in malnourished SSc patients in the median values of PTH (*p* = 0.02) and vitamin D levels (*p* = 0.008).
González-Martín et al., 2020 [14]	Cross-sectional study	70 patients diagnosed with SSc (diffuse or limited forms)	Lower levels of vitamin D in 59% of the SSc patients. Inverse association between serum vitamin D levels and carotid intima-media thickness (*p* = 0.025).
Horváth et al., 2019 [15]	Case-control study	44 SSc patients and 33 healthy controls	Vitamin D deficiency in SSc patients (*p* = 0.003).
Caimmi et al., 2019 [16]	Retrospective longitudinal study	65 SSc patients	Negative association between vitamin D levels and the risk of digital ulcers developing (*p* = 0.018). No significant differences in vitamin D between SS with or without vitamin D supplementation (*p* = 0.922). No significant differences in vitamin D variations for disease subset (*p* = 0.728), disease activity (*p* = 0.463), previous digital ulcers (*p* = 0.379), incident pulmonary arterial hypertension (*p* = 0.646), delta of body mass index (*p* = 0.824), delta of forced vital capacity (*p* = 0.633) or diffusion capacity of carbon monoxide (DLCO) (*p* = 0.647), smoking habit (*p* = 0.333), modified Rodnan skin score (mRSS) (*p* = 0.295).
Di Liberto et al., 2019 [17]	Prospective case-control study	45 SSc patients and 35 controls	The treatment with 1,25(OH)(2)D of regulatory T cells increased the production of IL-10, a cytokine able to modulate immune response (*p* < 0.0001).
Corallo et al., 2019 [18]	Prospective study	62 SSc Caucasian patients	No association between serum vitamin D levels and sarcopenia (*p* = 0.3).
Gupta et al., 2018 [19]	Pilot study	38 SSc patients, 38 controls	Significantly lower serum vitamin D levels in SSc patients compared with healthy controls (*p* = 0.001). No correlation between serum vitamin D levels and age, gender, disease duration or its variants, type of autoantibodies, presence of digital ulceration, or systemic involvement. Inverse correlation between serum vitamin D levels and mRSS (*r* = −0.267).
Taylan et al., 2018 [20]	Cross-sectional study	46 SSc patients and 30 healthy controls	Significantly lower vitamin D levels in SSc patients (*p* < 0.05).
Kotyla et al., 2018 [21]	Case-control study	48 patients with diffuse systemic sclerosis and 23 controls	A weak correlation between vitamin D levels and iFGF23 (*p* < 0.05). No association between vitamin D levels and the extent of skin involvement or disease activity (*p* = 0.97).
Trombetta et al., 2017 [22]	Retrospective study	154 SSc patients	A significant correlation of vitamin D levels with lung involvement (*p* = 0.04), peripheral vascular (*p* = 0.03), kidney (*p* = 0.02), and gastrointestinal damage (*p* = 0.05) and with seasonality (*p* = 0.0086) in SSc patients. Correlation between 25(OH)D and calcium serum concentrations (*p* = 0.05). No statistically significant correlation between 25(OH)D and gender (*p* = 0.63), age (*p* = 0.81), Raynaud's phenomenon duration (*p* = 0.69), disease duration (*p* = 0.43), dcSSc, lcSSc (*p* = 0.49). No significant correlations between digital ulcer incidence and 25(OH)D serum concentrations (*p* = 0.13).
Ahmadi et al., 2017 [23]	Case-control study	60 SSc patients (30 diffuse scleroderma and 30 limited scleroderma), 30 age- and sex-matched healthy controls	Lower serum levels of vitamin D in the SSc patients compared with healthy controls (*p* < 0.001). No significant differences in serum vitamin D levels between dcSSc and lcSSc (*p* = 0.395).
An et al., 2017 [24]	Meta-analysis	554 SSc patients and 321 controls	Lower serum vitamin D levels in SSc compared with healthy controls, in dcSSc compared to lcSSc. No correlation between vitamin D deficiency and mRSS (*p* = 0.47), systolic pulmonary pressure (*p* = 0.72), gastrointestinal ulcer (*p* = 0.98), and pulmonary involvement (*p* = 0.99).
Hajialilo et al., 2017 [25]	Cross-sectional study	60 SSc patients and 60 healthy controls	Significantly lower vitamin D levels in SSc patients (*p* = 0.001). No significant correlation between 25(OH)D levels and the presence of calcinosis and positive results for autoantibodies.
Zhang et al., 2017 [26]	Case-control study	60 SSc patients and 60 healthy controls	Lower vitamin D levels (*p* < 0.001) in SSc patients compared with healthy controls. High ratio of pulmonary involvement in patients with vitamin D insufficiency.
Giuggioli et al., 2017 [27]	Case-control study	140 patients	Hypovitaminosis D associated with autoimmune thyroiditis (*p* = 0.008) and calcinosis (*p* = 0.057). Decreased 25(OH)D levels correlated with increased PTH (*p* < 0.0001).
Park et al., 2017 [28]	Pilot study	40 female SSc patients and 80 healthy controls	Vitamin D deficiency associated with digital ulcer (*p* = 0.012), but not with atherosclerosis or arterial stiffness (*p* > 0.05).
Cruz-Domínguez et al., 2017 [29]	Cohort study	220 SSc patients	Lower vitamin D levels in SSc with and without calcinosis. No association between vitamin D levels and calcinosis (*p* = 0.56).
Groseanu et al., 2016 [30]	Cross-sectional study	51 SSc patients	Positive correlation between decreased vitamin D levels and pulmonary fibrosis (*p* = 0.011) and low DLCO (*p* = 0.019). Negative correlation between vitamin D status and diastolic dysfunction (*p* = 0.033), digital contractures (*p* = 0.036), and muscle weakness (*p* = 0.015). Negative association between vitamin D supplementation and development of digital ulcers (*p* = 0.04).
Atteritano et al., 2016 [31]	Case-control study	40 patients with scleroderma and 40 healthy controls	Significantly lower serum vitamin D in SSc patients (*p* = 0.0003). Inverse correlation between vitamin D serum concentrations in SSc and systolic pulmonary artery pressure (*p* = 0.013). Significant correlation between vitamin D and PTH serum levels in SSc (*p* < 0.05). Significant association between vitamin D insufficiency and mRSS (*p* = 0.02).
Sampaio-Barros et al., 2016 [32]	Cross-sectional study	38 female patients with diffuse SSc	Lower levels of vitamin D in anti-Scl-70-positive compared to anti-Scl-70-negative SSc (*p* = 0.039). Positive correlation of vitamin D levels with weight (*p* = 0.041), BMI (*p* = 0.038), total femur BMD (*p* = 0.037), femoral neck BMD (*p* = 0.017), SF-36-Vitality (*p* = 0.017), SF-36-Social Function (*p* = 0.05), SF-36-Emotional Role (*p* = 0.049), and SF-36-Mental Health (*p* = 0.0006). Negative correlation between 25(OH)D and quality of life in dcSSc: HAQ-Reach (*p* = 0.044) and HAQ-Grip Strength (*p* = 0.042). Negative correlation between vitamin D levels and severe nailfold capillaroscopy alterations: diffuse devascularization (*p* = 0.029) and avascular areas (*p* = 0.033).
Carmel et al., 2015 [33]	Case-control study	54 SSc patients and 41 healthy controls	Positive correlation between IgM 25(OH)D antibodies and scleroderma (*p* = 0.002). No correlation between vitamin D antibodies and other autoantibodies, disease severity, or target organ damage.
Corrado et al., 2015 [34]	Case-control study	64 postmenopausal SSc patients and 35 healthy control postmenopausal women	Significantly lower 25(OH)D levels in dcSSc compared to the lcSSc (*p* < 0.001). A significant association between degree skin fibrosis and circulating levels of 25(OH)D (*p* < 0.05). No correlation between 25(OH)D levels and presence of anti-centromere or anti-topoisomerase I autoantibodies and the disease duration. No correlation between malabsorption and 25(OH)D levels.
Atteritano et al., 2013 [35]	Case-control study	54 SSc women and 54 postmenopausal controls	Significantly lower vitamin D levels in SSc patients (*p* < 0.001). Significant correlation between vitamin D and PTH levels in SSc (*p* < 0.001).
Rios Fernández et al., 2012 [36]	Case-control study	100 SSc patients and 100 controls	Lower levels of vitamin D in SSc from the north of Spain in comparison with those in south of Spain (*p* < 0.031). Low bone mineral density (BMD) in 86% SSc with low levels of vitamin D (<30 ng/ml) compared with 66.7% of those with normal levels (*p* = 0.073). Significant association between vitamin D level heart involvement (*p* = 0.012), positive antinuclear antibody (ANA) (*p* < 0.006), and low DLCO (*p* = 0.017).
ibn Yacoub et al., 2012 [37]	Case-control study	60 SSc patients and 60 controls	Very low levels of vitamin D (*p* = 0.001) in SSc patients compared with controls. Significant correlation between vitamin D levels and joint pain severity, immunological status, and BMD in lumbar spine and femoral neck.
Avouac et al., 2012 [38]	Cross-sectional study	71 women with SSc, 139 women with RA, and 227 healthy women	Low levels of vitamin D is a risk factor for fractures (*p* = 0.03).
Shinjo et al., 2011 [39]	Case-control study	10 patients with JoSSc and 10 healthy controls	High prevalence of 25(OH)D insufficiency in JoSSc (*p* = 0.04) and correlation with hip BMD (femoral neck and total femur: *p* = 0.004 and *p* = 0.02, respectively).
Arnson et al., 2011 [40]	Retrospective cohort study	327 European patients with SSc and 141 healthy controls	Lower serum vitamin D concentrations (*p* < 0.001) and inverse relationship with cutaneous tissue fibrosis (*p* = 0.02). A negative correlation between vitamin D concentration and patient age (*p* < 0.05).
Gambichler et al., 2011 [41]	Observational study	137 SSc patients	Lower vitamin D levels (*p* < 0.0001). No significant relationship between serum 25(OH)D levels and SSc subtypes, lung fibrosis, renal involvement, gastroesophageal reflux disease, digital ulcers, mRSS, ANA, age, sex, BMD, and therapy.
Seriolo et al., 2011 [42]	Case-control study	53 SSc female patients with SSc and 35 sex-, age-, and season-matched control	During winter: vitamin D insufficiency in 60% SSc compared with 38% matched controls (*p* < 0.001); lower average serum 25(OH)D levels among SSc compared with controls (*p* < 0.001). During summer: 64% SSc patients and 36% controls with vitamin D insufficiency (*p* < 0.001); 24% SSc vs. 12% control with vitamin D deficiency (*p* < 0.0001); lower average serum 25(OH)D levels among SSc compared with controls (*p* < 0.001).
Caramaschi et al., 2010 [43]	Prospective study	65 SSc patients	Association between patients with vitamin D deficiency and longer disease duration (*p* = 0.026), lower DLCO (0.014), higher estimated PAP (*p* = 0.037), higher values of erytrocyte sedimentation rate (ESR) (*p* = 0.01), and C-reactive protein (CRP) (*p* = 0.004) and with nailfold videocapillaroscopic pattern (*p* = 0.013) in comparison with patients with vitamin D insufficiency.
Rios Fernández et al., 2010 [44]	Cohort study	63 SSc patients	Lower vitamin D levels. No significant correlation between vitamin D levels and sPAP and the presence or absence of lung fibrosis (*p* > 0.05).
Calzolari et al., 2009 [45]	Case-control study	60 SSc patients and 60 matched controls	Lower levels of vitamin D in SSc patients compared with controls (*p* < 0.001). No significant association between vitamin D concentrations and disease features (lcSSc or dcSSc, gastrointestinal involvement, cutaneous ulcers, and joint involvement) and no correlation with mRSS. Vitamin D and serum C-telopeptide of type I collagen negatively correlated in SSc (*p* = 0.01). Vitamin D correlates with physical performance score assessed by the Medical Outcomes Study SF-36 (Short Form-36 questionnaire) (*p* = 0.08).
Vacca et al., 2009 [6]	Prospective study	156 consecutive SSc patients (90 from Northern France and 66 from Southern Italy)	Slight association between vitamin D and anti-centromere antibodies (*p* = 0.04). Significant negative correlation between low serum vitamin D levels and European Disease Activity Score (*p* = 0.04). No correlation with CRP (*p* = 0.67). Vitamin D deficiency associated with sPAP estimated by echocardiography (*p* = 0.004) and pulmonary fibrosis (*p* = 0.04). No associations between vitamin D deficiency and acroosteolysis, calcinosis, HAQ, or Medsger disease severity scores.
Braun-Moscovici et al., 2008 [46]	Retrospective study	134 Mediterranean SSc patients	Hypovitaminosis D significantly correlated with ethnicity (Arab origin) (*p* = 0.009). Statistically significant relationship between vitamin D and PTH (*p* = 0.01), but not between vitamin D and acroosteolysis (*p* = 0.447).
Hulshof et al., 2000 [47]	Double-blind, placebo-controlled trial	27 patients: 20 with morphea and 7 with SSc	No significant difference in SSc between the placebo and 1,25(OH)(2)D groups after 9 months of treatment in the skin score, esophageal body motility, and oral aperture. No significant change in S-PIIINP in serum samples of SSc patients after 1,25(OH)(2)D treatment.
Humbert el al., 1993 [48]	Open prospective, uncontrolled study	11 SSc patients	Vitamin D treatment improved significantly oral aperture and flexion index distance (*p* < 0.05) and skin extensibility (*p* < 0.01).

## Data Availability

The data supporting this review are from previously reported studies, which have been cited.
